# Sirtuins and Resveratrol in Cardiorenal Diseases: A Narrative Review of Mechanisms and Therapeutic Potential

**DOI:** 10.3390/nu17071212

**Published:** 2025-03-30

**Authors:** Caterina Carollo, Alessandra Sorce, Emanuele Cirafici, Giuseppe Mulè, Gregorio Caimi

**Affiliations:** Department of Health Promotion, Mother and Child Care, Internal and Specialistic Medicine, University of Palermo, 90127 Palermo, Italyemanuele.cirafici@community.unipa.it (E.C.); giuseppe.mule@unipa.it (G.M.);

**Keywords:** sirtuins, SIRTs, aging, CKD, CVD, cardiorenal syndromes, CRS, resveratrol

## Abstract

Aging is a very complex process, and it has been linked with Sirtuins. Sirtuin enzymes are a family of deacetylases that are related to caloric restriction and aging by modulating energy metabolism, genomic stability, and stress resistance. Up to now, seven sirtuins have been recognized. This narrative review aimed to analyze the literature produced between January 2005 and March 2025 to evaluate the role of sirtuins in chronic kidney disease and, as heart and kidney diseases are strictly interrelated, to explore their role in heart diseases and cardio-renal cross-talk. A reciprocal relationship between CKD and aging seems to exist since CKD may contribute to premature biological aging of different organ systems. SIRTs are involved in the pathophysiology of renal diseases; their activation can delay the progression of several renal diseases. Notably, an increasing number of studies linked SIRTs with different CVDs. SIRTs affect the production of mitochondrial reactive oxygen species (ROS) by modulating mitochondrial function. The imbalance of SIRT levels may increase the vulnerability to CVDs. SIRTs are involved in the pathophysiological mechanisms of HFpEF (heart failure with preserved ejection fraction) through different signaling pathways. Fibrosis is the linkage mechanism between the heart and kidney in the development of cardio-renal diseases. Current studies on sirtuins, resveratrol, and cardiorenal disease highlight their potential therapeutic benefits in regulating blood pressure, kidney function, lipid profiles, and inflammation, making them a promising area of investigation for improving cardiovascular and renal health outcomes. However, significant gaps remain. The limited availability of highly selective and potent sirtuin modulators hampers their clinical translation, as most existing compounds exhibit poor bioavailability and suboptimal pharmacokinetic properties.

## 1. Introduction

Aging is a highly complex and multifactorial process influenced by genetic, environmental, and cellular pathways, involving intricate molecular mechanisms that regulate cellular homeostasis, metabolic balance, and stress responses [1]. Aging is an extraordinarily complex and multifactorial process shaped by an interplay of genetic predisposition, environmental influences, and cellular pathways. It involves intricate molecular mechanisms that regulate cellular homeostasis, metabolic balance, and stress responses. In recent decades, extensive research has underscored the pivotal role of epigenetic modifications, mitochondrial dysfunction, telomere attrition, and chronic inflammation—commonly termed “inflammaging”—in driving age-related physiological decline [2]. These interconnected factors contribute to cellular senescence, impaired tissue function, and increased susceptibility to age-associated diseases.

Among the key molecular regulators of aging, sirtuins (SIRTs) have garnered significant attention. This conserved family of NAD+-dependent proteins plays a crucial role in cellular stress resistance, metabolic regulation, and genomic stability. Sirtuins are known to modulate gene expression, DNA repair, mitochondrial function, oxidative stress resistance, and autophagy—hallmark processes linked to longevity [3]. However, while their role in aging and associated diseases is well established, the relationship between sirtuins and the aging process is far more intricate than often portrayed.

Aging is not dictated by molecular mechanisms alone; rather, it emerges from a complex interaction between genetic predispositions, environmental exposures, and lifestyle choices [4]. Factors such as diet, physical activity, circadian rhythm regulation, and exposure to toxins or pollutants profoundly influence sirtuin activity. For instance, caloric restriction and certain bioactive compounds like resveratrol have been shown to activate SIRT1, promoting longevity-associated pathways [5]. Conversely, chronic stress, obesity, and metabolic disorders can downregulate sirtuin function, accelerating cellular aging and increasing the risk of age-related diseases such as cardiovascular disease, neurodegeneration, and chronic kidney disease [6,7].

Furthermore, sirtuins do not act in isolation; they operate within a vast regulatory network that includes insulin signaling, AMPK activation, and mTOR inhibition—pathways that collectively influence lifespan and health span. The interaction between sirtuins and epigenetic modifications further adds to this complexity, as changes in DNA methylation, histone acetylation, and chromatin remodeling shape cellular responses to aging [8]. Additionally, sirtuin-mediated mitochondrial maintenance and energy metabolism are closely linked to the availability of key metabolites such as NAD+, whose levels decline with age [9].

Given these multifaceted interactions, a more comprehensive approach to studying sirtuins should consider their dynamic interplay with genetics, environmental stressors, and lifestyle interventions. Understanding these relationships provides valuable insights into potential therapeutic strategies aimed at promoting healthy aging and mitigating the impact of age-related diseases.

Seven sirtuins, listed from one to seven, have been recognized so far. Each shows a catalytic domain (present in all sirtuins), and different N- and C-ends give each sirtuin specific biological features [10]. 

Sirtuins (SIRT1–SIRT7) exhibit distinct intracellular localization, with SIRT1, SIRT6, and SIRT7 primarily nuclear, SIRT2 cytoplasmic, and SIRT3–SIRT5 mitochondrial, influencing diverse cellular functions. Their activity is modulated by various activators such as NAD^+^, resveratrol, and Piceatannol, and inhibitors like nicotinamide, EX-527 and SirReal2 [11].

**Figure 1:**
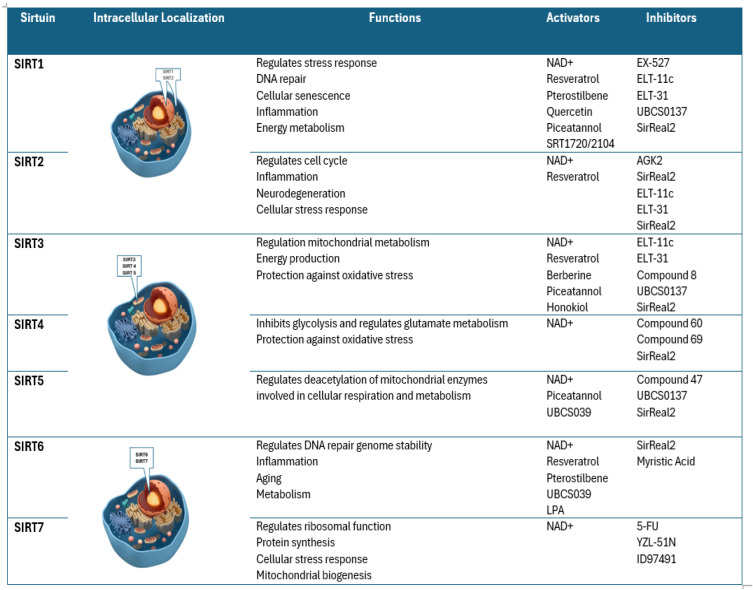
Main SIRTs characteristics: localization, functions, activators, and inhibitors [11].

Main SIRTs characteristics like function, intracellular localization, and respective activators and inhibitors are illustrated in Figure 1.

Sirtuins may promote different post-translational modifications in many different proteins, so they are actually known as deacetylases [12]. Sirtuins’ activity depends on nicotinamide adenine dinucleotide (NAD^+^). This is very important if we consider that NAD^+^ is a crucial cofactor for many mitochondrial metabolic processes that lead to energy production. Mitochondria are largely expressed in the heart and kidneys. Mitochondrial reactive oxygen species (ROS) production leads to altered DNA and electron chain transport, thus impairing mitochondrial function. This dangerous chain could be counteracted by healthy mitochondria and selective autophagy of damaged ones, thus explaining the beneficial effects of caloric restriction [13]. 

The classification of sirtuins as merely NAD^+^-dependent deacetylase is an oversimplification that does not fully capture the complexity of their biological roles. Although NAD^+^ is an essential cofactor for the majority of sirtuins, it is not the sole determinant of their enzymatic activity. Sirtuins are involved in a broader range of cellular processes and enzymatic modifications, extending well beyond deacetylation. This functional diversity underscores the need to recognize the multifaceted nature of sirtuin biology. While deacetylation is the most widely studied activity of sirtuins, some members of the sirtuin family catalyze additional post-translational modifications, including ADP-ribosylation [14] and deacetylation of non-acetyl groups [15]. Notably, SIRT5 is involved in the deacetylation of succinyl groups [16], highlighting the ability of sirtuins to modulate a diverse array of acyl groups beyond acetylation. Moreover, SIRT1 and SIRT6 have been shown to catalyze mono-ADP-ribosylation, a modification that plays a crucial role in DNA repair and various regulatory pathways, further broadening the scope of sirtuin function [17].

In our previous studies, we investigated the role of sirtuins in modulating factors that accelerate physiological aging, including glucose metabolism, DNA integrity, cancer development, neurodegenerative disorders, and chronic obstructive pulmonary disease (COPD) [18]. 

Building on our previous work and recent findings the current objective is to elucidate the role of sirtuins as key pathophysiological mediators and potential therapeutic targets in the progression of chronic kidney disease (CKD). Additionally, recognizing the intricate interrelationship between cardiovascular and renal dysfunction, we aim to investigate the involvement of sirtuins in heart diseases, with a particular focus on the cardio-renal axis and the molecular mechanisms underlying cardio-renal cross-talk.

## 2. Materials and Methods

This study utilizes a narrative review approach to provide a qualitative and interpretative synthesis of existing literature on the connection between sirtuins and cardiorenal disease. A comprehensive literature search was performed using Web of Science, PubMed, and Scopus, covering all articles indexed from January 2005 to March 2025. The search strategy incorporated keywords such as “sirtuins”, “aging”, “chronic kidney disease”, “cardiovascular disease”, “resveratrol”, and “cardiorenal syndrome”. After identifying the initial pool of records, three researchers (CC, AS, and MC) carried out the article screening process.

## 3. Sirtuins and Kidney Diseases

In kidney disease, sirtuins influence key pathological mechanisms such as fibrosis, apoptosis, mitochondrial dysfunction, and inflammation, which contribute to disease progression [19,20]. Dysregulation of sirtuin activity has been implicated in acute kidney injury [21], diabetic nephropathy [22], and chronic kidney disease [23,24]. Understanding the precise roles of sirtuins in renal physiology and pathology may provide new therapeutic targets for mitigating kidney disease and improving renal function. Regarding renal physiology, sirtuins’ expression in the kidney and their functions are various.

SIRT1 and SIRT6 play a crucial role in preserving both the structural and functional integrity of podocytes, which is essential for maintaining the filtration barrier in the kidneys. In addition to its role in podocyte health, SIRT1 is also involved in the regulation of endothelial function by modulating endothelial nitric oxide synthase (eNOS), which has a direct impact on systemic blood pressure [25].

SIRT3 contributes to vascular health by regulating vascular endothelial growth factor (VEGF), a key factor in maintaining endothelial integrity [26]. Within the proximal tubule, SIRT1, SIRT3, and SIRT5 are essential for mitochondrial function, ensuring that tubular cells produce adequate ATP to support solute reabsorption [27,28]. Meanwhile, in the distal tubules, SIRT1 plays a significant role in regulating sodium balance and water reabsorption by influencing the α-subunit of the epithelial sodium channel (ENaC) [29].

**Figure 2:**
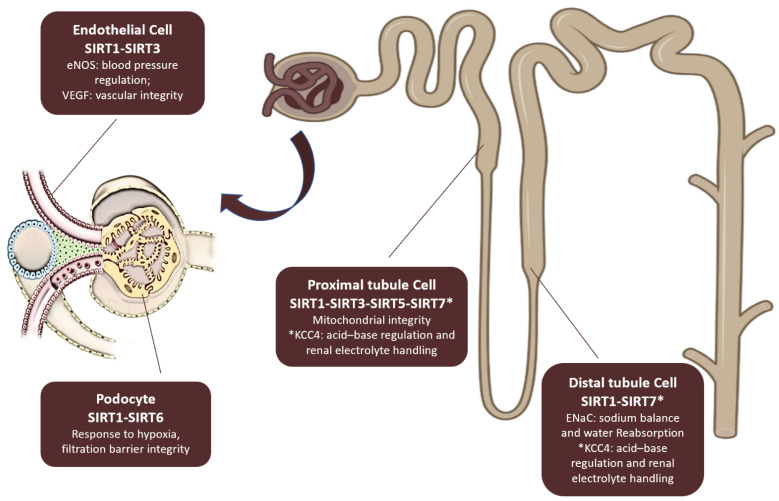
Sirtuins expression in both the glomerular and tubular compartments of the kidney. SIRT1 and SIRT6 are essential for the structural and functional integrity of podocytes, which helps maintain the filtration barrier. SIRT1 also regulates endothelial function by controlling endothelial nitric oxide synthase (eNOS), impacting systemic blood pressure. SIRT3 regulates vascular endothelial growth factor (VEGF), playing a role in endothelial integrity. In the proximal tubule, SIRT1, SIRT3, and SIRT5 preserve mitochondrial function, enabling tubular cells to generate the ATP required for solute reabsorption. In the distal tubules, SIRT1 is involved in regulating sodium balance and water reabsorption by controlling the α-subunit of the epithelial sodium channel (ENaC). In both proximal and distal tubules, SIRT7 regulates the acid–base balance and renal electrolyte handling by deacetylating the K+/Cl− cotransporter 4 (KCC4).

Moreover, in both the proximal and distal tubules, SIRT7 is involved in maintaining an acid–base balance and renal electrolyte homeostasis. This is achieved through the deacetylation of the K+/Cl− cotransporter 4 (KCC4), which is crucial for proper renal ion transport [30]. These findings highlight the diverse and essential functions of sirtuins in kidney physiology, particularly in maintaining cellular homeostasis and overall renal function. Sirtuins expression in both the glomerular and tubular compartments of the kidney are illustrated in Figure 2.

### 3.1. Sirtuins and Acute Kidney Injury

Aging kidneys show an increased vulnerability to ischemia/reperfusion (I/R)-induced injury, suggesting that sirtuins might play a significant role in the response to I/R. SIRT1 has been shown to confer resistance to kidney injury after I/R [31]. Overexpression of SIRT1 reduces kidney injury by activating antioxidant pathways such as the Nrf2/HO-1 signaling pathway and reducing p53 expression, which subsequently attenuates apoptosis [31,32]. Additionally, SIRT1 promotes mitochondrial biogenesis, restoring ATP levels and reducing mitochondrial mass, nitrosative stress, and inflammation, which help protect against I/R injury. Activators like SRT1720, which stimulate SIRT1, have been shown to improve renal function following I/R injury by enhancing mitochondrial function and reducing inflammation [33]. The activation of mitochondrial biogenesis through PGC-1α is proposed as a key mechanism for repair after I/R injury [34].

SIRT3, predominantly localized in the mitochondrial matrix, is another key sirtuin involved in I/R injury. Following I/R, SIRT3 expression increases, and its overexpression helps protect the kidneys by suppressing superoxide generation [35]. Loss of SIRT3, however, exacerbates kidney injury by impairing mitochondrial homeostasis, a process that can be rescued through restoration of SIRT3 via the AMPK/PGC-1α pathway [36].

Interestingly, SIRT5 has a distinct role in I/R injury, with loss of SIRT5 function conferring renoprotective effects. SIRT5 regulates fatty acid oxidation by shifting it from the mitochondria to peroxisomes, which appears to improve kidney function after I/R [37]. Conversely, SIRT6 has been linked to the negative regulation of tubular cell injury and inflammation during I/R injury, suggesting a protective role [38].

Sirtuins, particularly SIRT1 and SIRT6, have been found to alleviate kidney injury in sepsis-induced AKI by modulating inflammatory responses and promoting autophagy. In contrast, the role of SIRT2 in sepsis-induced AKI appears to be detrimental, as the absence of SIRT2 improves renal function and reduces tubular injury [39]. SIRT1 has also been shown to attenuate contrast-induced nephropathy (CIN), a common cause of AKI, through its modulation of oxidative stress and apoptosis via the PGC-1α/FoxO1 signaling pathway [40]. SIRT3 deficiency exacerbates CIN, while activation of the SIRT3-Nrf2 pathway provides protection [41].

### 3.2. Sirtuins in Chronic Kidney Disease and Fibrosis

By 2021, a joint statement from the American Society of Nephrology, the European Renal Association, and the International Society of Nephrology indicated that more than 850 million people suffer from some form of kidney disease. CKD affects between 8% and 16% of the population worldwide. It is defined by a glomerular filtration rate (GFR) of less than 60 mL/min/1.73 m^2^, albuminuria of at least 30 mg per 24 h, or markers of kidney damage (e.g., hematuria or structural abnormalities such as polycystic or dysplastic kidneys) persisting for more than 3 months [42].

CKD is associated with a great increase in morbidity and mortality and a decrease in health-related quality of life. The severity of these complications is generally proportional to the decline in renal function, and it is most evident in patients with end-stage renal disease (ESRD) [43]. Moreover, CKD causes very important economic consequences. In 2023, in the USA, there was a 40% increase, from USD54.9B to USD76.8B, in expenditures for individuals with CKD [44].

The risk of CKD increases with age, and elderly patients are overrepresented in the dialysis population [45]. Moreover, a reciprocal relationship between CKD and aging seems to exist since CKD may contribute to premature biological aging of different organ systems [46] This may result in the occurrence of usually geriatric complications in relatively young patients with ESRD [16]. This phenomenon, referred to as “premature aging”, is due to the interplay of various biological processes and signaling pathways that drive both renal injury and systemic age-related dysfunction. One of the primary mechanisms by which CKD accelerates aging is through the enhanced production of reactive oxygen species (ROS) and chronic inflammation. Oxidative stress plays a central role in aging and is a key driver of tissue damage in CKD. Elevated ROS levels contribute to endothelial dysfunction, inflammation, and fibrosis, which in turn promote the premature aging of organs beyond the kidney [47,48]. Telomere shortening is another hallmark of aging and is accelerated in CKD patients. Shortened telomeres are associated with cellular senescence, a state of irreversible cell cycle arrest that contributes to tissue dysfunction and aging. In CKD, the process of cellular senescence is observed in renal cells as well as in other tissues, leading to impaired tissue repair, increased fibrosis, and functional decline [49,50]. Mitochondrial dysfunction is a key feature of both aging and CKD. In CKD, mitochondrial damage results from increased oxidative stress, altered mitochondrial dynamics (such as mitochondrial fission), and impaired mitophagy, leading to a decline in cellular energy and function [51]. This mitochondrial dysfunction is particularly evident in CKD-related complications such as cardiovascular disease and skeletal muscle wasting, which are often seen in elderly CKD patients [52]. Endothelial dysfunction is a critical factor in the accelerated aging process in CKD. In CKD, endothelial cells become dysfunctional due to oxidative stress, inflammation, and impaired nitric oxide signaling, leading to vascular stiffness and impaired organ perfusion [53]. This is especially relevant in elderly CKD patients who are at higher risk of developing atherosclerosis and other cardiovascular complications associated with aging.

Fibrosis is a hallmark of nearly all progressive forms of chronic kidney disease (CKD), regardless of the initial cause [54]. This fibrotic progression is closely linked to impaired NAD+ biosynthesis, which downregulates the activity of sirtuins, including those with anti-fibrotic roles. In rat models of CKD, it has been shown that NAD+ biosynthesis is significantly reduced, impairing the functions of SIRTs, which are crucial for combating fibrosis [55].

Each of the seven sirtuins plays a role in this complex relationship.

Sirtuins (SIRT1, SIRT3, and SIRT6) exert anti-fibrotic effects by modulating key molecular pathways, including TGFβ/Smad, Wnt/β-catenin, Notch, and NF-κB. The TGFβ (transforming growth factor-β) pathway is the primary driver of fibrosis in CKD. When activated, TGFβ binds to its receptors (TGFβR1 and TGFβR2), leading to the phosphorylation and activation of Smad2/3, which then forms a complex with Smad4 and translocates to the nucleus to promote the transcription of pro-fibrotic genes, such as collagen I, fibronectin, and matrix metalloproteinases (MMPs) [56]. The Wnt/β-catenin pathway plays a critical role in kidney fibrosis by regulating epithelial-to-mesenchymal transition (EMT), a process where renal epithelial cells lose their polarity and acquire a fibroblast-like phenotype, contributing to ECM accumulation [57]. The Notch pathway is another critical regulator of kidney fibrosis, involved in tubulointerstitial fibrosis and glomerulosclerosis. Upon ligand binding, Notch intracellular domains (NICDs) translocate to the nucleus and activate pro-fibrotic genes [58]. Inflammation is a major contributor to kidney fibrosis, and the NF-κB pathway is a key regulator of inflammatory responses in CKD [59].

SIRT1 expression is notably decreased in kidney biopsies from patients with focal segmental glomerulosclerosis (FSGS) [60]. Experimental studies have shown that silencing SIRT1 exacerbates fibrosis in mice with unilateral ureteral obstruction (UUO), a model of progressive kidney injury [61]. In contrast, stimulating SIRT1 through pharmacological interventions or genetic manipulation has been found to reduce inflammation and matrix protein accumulation, thus mitigating fibrosis in experimental models of CKD [62].

The anti-fibrotic activity of SIRT1 is primarily attributed to its ability to inhibit the TGFβ signaling pathway by deacetylating Smad3 and Smad4. This action reduces the transcription of pro-fibrotic genes such as collagen IV, fibronectin, and matrix metalloproteinase 7 (MMP7) [63]. Additionally, SIRT1 influences endothelial cell function, as demonstrated by targeted deletion of SIRT1 in the endothelium, which impaired vasorelaxation and promoted fibrosis by downregulating MMP14, stimulating the HIF2α–Notch1 axis, and releasing proteolytic fragments of the endothelial glycocalyx [64].

SIRT6 also plays a protective role against kidney fibrosis. After fibrotic insults, SIRT6 is upregulated in the kidneys and interacts with β-catenin. This complex binds to the promoters of fibrogenic genes and prevents their transcription through SIRT6-dependent deacetylation of histone proteins [65]. SIRT6 overexpression in mice prevented renal interstitial fibrosis induced by a high-adenine diet by downregulating HIPK2, which regulates several pro-fibrotic pathways [66]. In renal tubular cells, the phosphorylation of SIRT6 by GSK3β prevented its degradation, thus supporting its anti-fibrotic actions. SIRT3, a mitochondrial sirtuin, has been shown to play a role in mitigating fibrosis in multiple organs, including the kidney. In SIRT3-deficient mice, age-dependent fibrosis in the kidney is exacerbated due to increased TGFβ signaling and hyperacetylation of GSK3β, which leads to the activation of Smad3 [67]. Additionally, SIRT3-deficient mice exhibited reduced levels of mitochondrial fusion proteins (Opa1 and Mfn1) and increased levels of Drp1, leading to mitochondrial fission, a process associated with renal dysfunction and fibrosis since its early stage [68].

In podocytes, SIRT3 has been shown to deacetylate KLF15, a negative regulator of extracellular matrix protein synthesis, helping to reduce fibrosis [69]. Moreover, SIRT3 inhibits renal calcium oxalate crystal formation by promoting macrophage M2 polarization via the deacetylation of FOXO1 [70] and protects from hyperlipidemia-related renal injury [71].

### 3.3. Sirtuins and Hypertensive Nephropathy

Sirtuins, particularly SIRT3 and SIRT6, have been shown to exert protective effects in the pathogenesis of hypertensive nephropathy through various biological functions such as antioxidative stress, anti-apoptosis, anti-fibrosis, anti-inflammation, and anti-mitochondrial injury. The protective mechanisms of these sirtuins are primarily achieved by regulating protein post-translational modifications (PTMs), signaling pathways, and transcription factors. Studies using SIRT3 knockout or overexpression mouse models have demonstrated that SIRT3 plays a critical role in alleviating renal fibrosis and oxidative stress in hypertensive nephropathy. In SIRT3-deficient mice receiving Angiotensin II (Ang II) infusion, there was an increase in endothelial mesenchymal transformation (EndoMT) and reactive oxygen species (ROS), which aggravated renal dysfunction [72]. On the other hand, SIRT3 transgenic endothelial-specific mice (TgEC) exhibited alleviation of Ang II-induced renal fibrosis, EndoMT, and oxidative stress, indicating that SIRT3 plays a protective role in mitigating hypertension-induced kidney damage [73]. SIRT3 knockout also led to iron overloading and enhanced ROS formation in renal cells via NADPH oxidase, further worsening renal fibrosis; in contrast, SIRT3 overexpression protected against kidney injury induced by Ang II [69,74].

SIRT6 also plays a key protective role in hypertensive nephropathy by regulating protein PTM, DNA damage, cellular metabolism, and mitochondrial function. In endothelial cells, SIRT6 prevents injury by inhibiting the NK3 homeobox 2-GATA binding protein 5 signaling pathway through deacetylation of histone H3 lysine 9 (H3K9) [75]. Furthermore, SIRT6 has been shown to alleviate Ang II-mediated inflammation and oxidative damage induced by Ang II suppressing ROS and enhancing gene expression of Nrf2 and HO-1 [76].

### 3.4. Sirtuins and Diabetic Kidney Disease

One of the most common causes of CKD is diabetes. Diabetic kidney disease (DKD) is defined by the presence of CKD in a person with diabetes [30], and it is considered to be the leading cause of end-stage renal disease (ESRD) in developed and developing countries [77]. Sirtuins play a critical role in the pathogenesis of DKD through their regulatory effects on various cellular pathways [78]. Among them, SIRT1, SIRT3, SIRT4, SIRT6, and SIRT7 have been demonstrated to exert renoprotective effects during both the initiation and progression of DKD [79,80,81]. These protective actions are mediated through their ability to deacetylate key target proteins and directly modulate the expression of genes and signaling pathways. In patients with DKD, SIRT1 expression is significantly downregulated in both serum and renal tissues, suggesting a strong correlation between its expression levels and kidney function [82]. SIRT1 exerts its protective effects by regulating transcription factors such as NF-κB and FoxO, thereby reducing inflammation, oxidative stress, and apoptosis; it also influences key signaling pathways, including AMPK/SIRT1, which promotes autophagy and mitochondrial function, and TGF-β1/Smad3, which mitigates fibrosis [83]. Furthermore, SIRT1 interacts with molecules like NQO1 and glucagon-like peptide-1 to prevent apoptosis and oxidative stress, ultimately alleviating DKD progression [84]. SIRT3 alleviates DKD by preventing HG-induced apoptosis, reducing ROS production, and activating autophagy through AMPK/PGC-1α signaling [85]. SIRT4 protects podocytes by reducing mitochondrial ROS and inhibiting NF-κB-mediated inflammation and apoptosis [86]. SIRT7 mitigates podocyte apoptosis and inflammation under diabetic conditions [87]. Collectively, these sirtuins contribute to mitochondrial homeostasis and anti-inflammatory mechanisms, highlighting their therapeutic potential in DKD. The roles of SIRT2 and SIRT6 in DKD remain unclear due to conflicting findings. SIRT2 expression is reported to be both upregulated and downregulated under hyperglycemia, with its knockdown enhancing cell proliferation and reducing apoptosis, suggesting a potential role in DKD pathology; meanwhile, SIRT6 is linked to increased TNF-α expression, suggesting a role in inflammation [22,88,89].

### 3.5. Sirtuins and Polycystic Kidney Disease

The role of SIRTs in autosomal-dominant polycystic kidney disease (ADPKD) is still emerging. Studies show that SIRT1 inhibition—either through silencing or pharmacological means—reduces cyst formation in mice. This negative effect of SIRT1 in ADPKD is linked to its suppression of Rb and p53, which promotes epithelial cell proliferation and cyst growth [90]. However, a clinical trial testing oral niacinamide, a SIRT inhibitor, in ADPKD patients showed good safety and tolerability but failed to slow kidney enlargement over 12 months [91]. In ADPKD patients compared to healthy subjects, Kurtgoz and coworkers found that urine SIRT1 levels were significantly lower. In addition, serum SIRT1 levels of ADPKD patients were higher than control cases, but the difference was not statistically significant. These findings suggest an impaired SIRT1 metabolism in ADPKD patients, which might play a role in cysts development [92]. SIRT2 is involved in cilia pathophysiology and centrosome function that in turn are involved in polycystic kidney disease and ciliopathy-associated disease progression [90]. Moreover, by inhibiting caspase-3 and ROS generation, it affects apoptosis and oxidative stress [93].

## 4. Sirtuins and Cardiovascular Diseases

Cardiovascular diseases (CVDs), principally ischemic heart disease (IHD) and stroke, are the leading cause of global mortality and a major contributor to disability. The total number of cardiovascular events almost doubled from 1990 to 2019 [94]. Notably, an increasing number of studies linked SIRTs with different CVDs. SIRTs affect the production of mitochondrial reactive oxygen species (ROS) by modulating mitochondrial function and increasing endothelial dysfunction, leading to an increased progression of atherosclerotic lesions [95]. The imbalance of SIRT levels may increase the vulnerability to CVDs, including heart failure (HF), atherosclerosis, ischemic heart disease, hypertrophic heart disease, and metabolic disease.

It seems that SIRT1 protects the heart from hypertrophic stimulation, oxidative stress damage, ROS accumulation, and apoptotic damage. It also could avoid ischemic/reperfusion injury, as well as SIRT3 and SIRT6 [96,97,98]; SIRT3 exerts a cardioprotective role by protecting mitochondrial function; evidence suggests that activation of SIRT6 may be a therapeutic tool to treat atherosclerosis.

The role of SIRTs in atherosclerosis is relatively clear, with main effects in regulating LDL cholesterol levels, macrophages, foam cells, and endothelial function through various factors and signaling pathways; SIRT1, SIRT3, and SIRT6 are the sirtuins involved in protecting against atherosclerosis and cardiac hypertrophy [99,100,101]. They also exhibit a protective role in diabetic cardiomyopathy. Unfortunately, not all SIRT activities are beneficial. For example, SIRT2 is destructive in I/R injury (its downregulation is protective against I/R injury [102]), and SIRT4 is detrimental to heart hypertrophy and fibrosis [103].

SIRTs are involved in the pathophysiological mechanisms of HFpEF (heart failure with preserved ejection fraction) [104]. As summarized by the European Society of Cardiology [105], it is the most significant subtype of HF, and it is primarily characterized by left ventricular diastolic dysfunction (LVDD).

The SIRT family has recently been found to be associated with HFpEF development through different signaling pathways such as the eEF2K/eEF2 pathway, the SIRT1/transmembrane BAX inhibitor motif, the Sirt3/MnSOD pathway, and the AMPK/PGC-1α pathway, causing endoplasmic reticulum stress, apoptosis, mitophagy, oxidative stress, and mitochondrial dysfunction [106]. SIRT4, SIRT5, and SIRT7, instead, are downregulated in HF.

## 5. Sirtuins and Cardio-Renal Diseases

It is well known that fibrosis is also involved in the onset of HF, and it is also recognized as the linkage mechanism between the heart and kidney in the development of cardio-renal diseases. Fibrosis arises from the proliferation of fibroblasts and their differentiation to myofibroblasts and subsequent deposition of extracellular matrix (ECM). Another important factor involved in heart and kidney fibrosis is the endothelial-to-mesenchymal transition (EndMT) [107,108], which seems to be an important mechanism leading to glomerular sclerosis in DKD [109]. Several studies have shown that TGF-β, through activation of several signaling pathways (TGF/Smad, Erk, Akt), is the main stimulator and modulator of EndMT [110]. According to previous studies, SIRT1 and SIRT3 (both decreased in TGF-β-induced EndMT) seem to be TGF-β inhibiting factors [111,112], so their upregulation could be a chance to attenuate both cardiac and renal fibrosis, thus mitigating cardio-renal syndromes, in which fibrosis plays an important role [113].

One of the most important recognized mechanisms leading to cardiorenal syndromes (CRS) is endothelial dysfunction [114,115,116]; indeed, it plays an important role in the pathophysiology of hypertension, which is considered to be one of the most common risk factors in the development of both renal and cardiac damage. Guo J et al., in a murine model, demonstrated that the downregulation of SIRT6 contributes to endothelial dysfunction and is involved in the pathogenesis of hypertension. The aim of the study was to explore the role of SIRT6 in the development of hypertension and the molecular mechanisms involved. It was found that SIRT6 indirectly regulates the expression of GATA5, a transcription factor that regulates blood pressure, by inhibiting Nkx3.2 expression. It is clear that the downregulation of SIRT6 reduces GATA5 expression, and this leads to endothelial dysfunction through reduced nitric oxide bioavailability, increased permeability, and subsequent hypertension and cardiorenal injury. These findings suggest that SIRT6 upregulation may be useful to counteract both hypertension and cardiorenal damage by improving endothelial function.

Mitochondrial dysfunction is another important factor involved in the pathophysiology of cardiorenal syndromes; it entails the reduction of glutathione (GSH) levels, increased inflammation, and altered redox signaling, all of these factors are involved in the genesis of oxidative stress and subsequential cardiorenal damage. The SIRTs’ role in mitochondrial impairment emerges from the involvement of SIRT1 and SIRT3 in the AMPK-SIRT1/3-PGC-1α axis in CRS 3 and 4, in which a primary acute or chronic kidney damage leads to cardiac dysfunction. This axis regulates several mitochondrial pathways, such as fatty acid oxidation, oxidative phosphorylation (OXPHOS), inflammation, and mitochondrial ROS production. It was found that the AMPK-SIRT1/3-PGC-1α axis is downregulated in CRS 3 and 4, and it is closely related to mitochondrial impairment; moreover, it seems that N-acetylcysteine (NAC) has protective effects against oxidative stress and inflammation thanks to its ability to increase AMPK, SIRT1, and SIRT3 expression. In conclusion, the use of antioxidants (such as NAC) should be considered as a strategy to reduce cardiorenal damage through the restoration of the AMPK-SIRT1/3-PGC-1α axis activity, exploiting its ability to mitigate both inflammation and oxidative stress by increasing the expression of SOD2 (superoxide dismutase) and GPx (glutathione peroxidase) and decreasing NLRP3 inflammasome/IL-1β/IL-18 activation [117].

## 6. Resveratrol and Cardio-Renal Diseases

After this dissertation, it is clear how sirtuins deserve our attention for their deep involvement in metabolic and pathophysiological ways of kidney and heart disease so these enzymes could be hypothesized as therapeutical targets. Different natural SIRT1 agonists showed renoprotective effects in animal models [118], and among these, resveratrol (RSV) and other phenolic compounds have been widely investigated with interesting and positive results.

Polyphenols, a diverse group of phytochemicals found in various fruits, vegetables, teas, and wines, have garnered significant attention in recent years for their potential health benefits, particularly in the context of cardiorenal diseases. These compounds have been shown to exhibit antioxidant, anti-inflammatory, and vasodilatory effects, which may be beneficial for cardiovascular and renal health.

RSV has been proven to be useful in treating different chronic diseases through enhancing mitochondrial quality [119], which is altered in several chronic diseases, such as Alzheimer’s disease (AD), Parkinson’s disease (PD), cardiovascular disease, obesity, cancer, and various forms of CKD (including DKD, IgA kidney disease, membranous nephropathy, and polycystic kidney disease) [120]. RSV seems to be able to regulate mitochondrial dynamics, mitophagy, endogenous mitochondrial apoptosis, oxidative stress, mitochondrial membrane homeostasis, respiratory chain function, and mitochondrial quality control, thus contributing to the delay of the onset of the above-mentioned chronic diseases [119]. SIRT1 activated by RSV attenuates ISO-induced cardiac dysfunction and fibrosis by regulating EndMT in vivo [121]. SIRT1 overexpression suppressed the development of TGF-β1-induced EndMT in vitro. In this study, it was also shown that P-Smad2/3 expression was increased in ISO-induced cardiac fibrosis but was attenuated by RSV-activated SIRT1 (perhaps due to SIRT1’s ability to inhibit the nuclear translocation of Smad2/3).

In addition, in CKD patients, resveratrol exerts beneficial, promising effects by modulating SIRT1 levels, oxidative stress, and inflammation.

Nearly 250 clinical trials involving resveratrol are currently in progress or have been completed, exploring its effects across various medical conditions [122]. However, in the majority of cases where resveratrol has been evaluated, the treatment has not demonstrated significant therapeutic benefits, often yielding neutral outcomes. While preclinical studies suggest promising biological effects, translating these findings into clinical success remains a challenge.

There is substantial variability in study results regarding resveratrol as a natural SIRT1 activator, which can be attributed to several factors. The transition of SIRT modulators from research to clinical application has been challenging due to factors such as insufficient selectivity for specific SIRT isoforms, low potency, poor bioavailability, and suboptimal pharmacokinetic and pharmacodynamic properties, as well as differences in clinical trial design and the baseline metabolic characteristics and dietary factors of the studied populations.

The existing human clinical trials on resveratrol have primarily focused on its safety profile and bioavailability, consistently indicating that it is well-tolerated but exhibits low bioavailability. However, only a limited number of studies have investigated whether resveratrol can replicate the physiological benefits observed in preclinical models [123].

Targeting SIRTs for kidney disease treatment holds significant potential, particularly in addressing age-related renal conditions. This is evident from the effects of SIRT-activating compounds (STACs) in experimental settings. Several small-molecule compounds, including SRT1720, SRT2104, SRT2183, and SRT3025, have demonstrated the ability to enhance SIRT1 activity and mitigate renal damage in preclinical models [106,123,124]. Among them, SRT2104 and SRT3025 have advanced to clinical trials, reflecting their promise as therapeutic agents. SRT2104, a selective SIRT1 activator, has shown beneficial effects in metabolic disorders, cardiovascular health, and inflammatory diseases, in addition to its renal-protective properties [124,125]. SRT3025, another SIRT1 activator, has been explored for its role in promoting endothelial function and reducing fibrosis, which are key factors in kidney disease progression [126,127].

## 7. Sirtuins Agonists in Cardiorenal Disease

Sirtuins agonists (represented by NAD^+^) have gradually emerged as new treatments for heart failure. By modulating metabolism, maintaining redox homeostasis, and regulating immune responses, sirtuins improve heart failure symptoms and prognosis.

More recently, liraglutide, a novel antidiabetic agent, was shown to influence SIRT1 expression in stroke patients, and this finding confirms that sirtuins are worthy of investigation to ameliorate treatment and prognosis of CKD patients, independently of the cause of primary kidney disease [128].

SGLT2 inhibitors have been demonstrated to upregulate SIRT3 expression. SGLT2 inhibition suppressed epithelial-to-mesenchymal transition and fibrogenesis in kidney proximal tubules; this effect of SGLT2 inhibition was associated with its ability to restore SIRT3 expression and glycolysis [129]. This interesting pharmacological activity could be a potential therapeutical target to prevent cardiac remodeling. Moreover, SGLT2 inhibitors activate SIRT1/PGC-1α/FGF21 pathway through their ability to induce a fasting-like metabolic and transcriptional paradigm [130]. The cardioprotective effects of SGLT2 inhibitors may also be related to this particular signaling [131].

## 8. Future Directions

Future research is obviously needed to clarify the role of these molecules and to enhance their potential therapeutical role in heart and kidney diseases. However, in our opinion, this topic is worthy of more attention because resveratrol or other sirtuins agonists’ administration could become a useful and safe tool in cardiorenal diseases.

In diabetic rats, the intergenerational treatment with oral resveratrol improved the functions of the heart, kidney, and brain. The more interesting finding is that resveratrol treatment increases the second and third generations’ resistance to neurobehavioral changes, diabetes, and associated cardio-renal dysfunction [132].

It is really interesting to hypothesize similar studies in humans to prove the efficacy and safety of this natural compound.

A systematic review and meta-analysis analyzed multiple studies on polyphenol intake and kidney health. The review concluded that higher dietary polyphenol intake was associated with a lower risk of chronic kidney disease (CKD) and improved renal function markers among the general population [133]. This aligns with findings from another study [134], which demonstrated that higher consumption of flavonoid-rich foods was inversely related to the incidence of CKD in older adults.

In addition to their effects on blood pressure and kidney function, the anti-inflammatory properties of polyphenols have been shown to play a crucial role in protecting against the progression of cardiorenal diseases. A recent study [135] explored the impact of berry polyphenols on inflammatory markers in patients with chronic heart failure. The findings suggested that berry supplementation led to a significant reduction in inflammatory cytokines and improved cardiac function, highlighting the protective role of polyphenols in cardiovascular health [136].

## 9. Conclusions

In summary, emerging research underlines the potential of polyphenols as a promising dietary intervention for the prevention and management of cardiorenal diseases. Current research on sirtuins, resveratrol, and cardiorenal disease highlights their potential therapeutic benefits on blood pressure, kidney function, lipid profiles, and inflammation, making them a valuable area of study for improving cardiovascular and renal health outcomes but also revealing significant gaps. The lack of highly selective and potent sirtuin modulators limits their clinical translation, as most available compounds exhibit poor bioavailability and suboptimal pharmacokinetics. Moreover, the precise mechanisms underlying resveratrol’s cardioprotective and renoprotective effects remain incompletely understood, particularly regarding its interaction with different sirtuin isoforms. Further large-scale clinical trials and mechanistic studies are needed to clarify these aspects and optimize therapeutic strategies.
